# Wearable Electrohysterography Systems: A Systematic Review of Electrode Topology, Design Considerations, and Clinical Feasibility

**DOI:** 10.3390/s26206539

**Published:** 2026-10-15

**Authors:** Ressy Jaya Yanti, Eiji Kondoh, Tomohiko Igasaki

**Affiliations:** 1 Graduate School of Science and Technology, Kumamoto University, Kumamoto 860-8555, Japan; 2 Faculty of Life Sciences, Kumamoto University, Kumamoto 860-8556, Japan; 3 Faculty of Advanced Science and Technology, Kumamoto University, Kumamoto 860-8555, Japan

**Keywords:** uterine activity, labor progression, electrohysterography, electrode topology, wearable integration, clinical feasibility

## Abstract

**Highlights:**

This study provides a critical synthesis of the evolution of wearable electrohysterography (EHG), highlighting a fundamental design trade-off between spatial resolution and clinical feasibility. Current evidence indicates that electrode configuration requires a practical trade-off between spatial characterization and implementation complexity in wearable EHG monitoring. The findings highlight the importance of considering electrode configuration, acquisition approaches, wearable implementation, and signal processing requirements together when developing wearable EHG monitoring systems.

**What are the main findings?**

- The evolution from wired to patch-based and wearable EHG systems highlights the interplay among electrode density, spatial coverage, signal fidelity, robustness, and usability.
- Wearable EHG systems require coordinated optimization of electrode topology, signal acquisition, and wearable integration, with 8–16 electrodes representing a practical range for balancing spatial coverage and usability; however, challenges related to standardization, motion artifacts, and electrode–skin interface stability remain.

**What are the implications of the main findings?**

- Technical Implications: Highlights practical considerations for electrode configuration, signal acquisition, and wearable implementation.
- Clinical Implications: Identifies usability, signal reliability, and validation priorities relevant to the future clinical implementation of wearable EHG monitoring.

**Abstract:**

Electrohysterography (EHG) is an advanced, noninvasive technique for characterizing myometrial activity from the maternal abdominal surface. Despite its clinical relevance, the adoption of EHG in wearable systems is hindered by variability in electrode configurations and a lack of integrated system design. A PRISMA 2020-based systematic review of publications from September 1997 to January 2026 was conducted using IEEE Xplore, PubMed, Semantic Scholar, ScienceDirect, SpringerLink, MDPI, IOP, and Frontiers, supplemented by Crossref searches via Publish or Perish and manual citation tracking. Of the 1084 records identified, 91 publications were included in the evidence synthesis. The analysis examined electrode configurations, wearable design, and measurement methodologies, focusing on the effects of electrode density and placement on signal quality and clinical usability. The reviewed literature indicates that configurations employing approximately 8–16 electrodes represent a practical range for balancing spatial characterization and clinical usability. However, the findings reveal persistent challenges related to the limited integration of electrode design, signal acquisition, and analytical methods. This evidence underscores the need for an integrated approach that considers flexible electrode interfaces, standardized electrode configurations, and adaptive signal processing techniques. By aligning technological improvements with clinical requirements, this study identifies practical considerations for developing wearable maternal–fetal monitoring systems.

## 1. Introduction

The accurate assessment of uterine activity (UA) is essential in obstetric care, as uterine contractions (UCs) are the primary drivers of labor progression and influence clinical decision-making throughout pregnancy and childbirth [1,2]. Reliable evaluation of UA is crucial not only for the diagnosis and management of preterm labor but also for intrapartum care at term, including labor induction and augmentation, monitoring of labor progress, and assessment of contraction effectiveness. Preterm birth, defined by the World Health Organization (WHO) as delivery before 37 completed weeks of gestation, affects approximately 15 million pregnancies annually worldwide, representing nearly 10% of all births [1,3].

UCs serve as key indicators of UA and are fundamental for assessing labor progress and obstetric risk [4]. However, current clinical monitoring techniques have significant limitations. Tocodynamometry (TOCO), the most widely used noninvasive method for monitoring UCs, is susceptible to motion artifacts, maternal obesity, and transducer displacement [5,6,7,8,9,10]. The intrauterine pressure catheter (IUPC), considered the reference standard for direct measurement of intrauterine pressure, is invasive and poses risks such as infection and uterine injury [5,11]. Moreover, both TOCO and IUPC primarily measure the mechanical aspects of UA and provide limited insight into the underlying electrophysiological processes of labor. Consequently, these methods have limited ability to determine whether observed contractions are physiologically effective and contribute to labor progression.

Electrohysterography (EHG) has emerged as a promising noninvasive modality for monitoring UA and predicting the onset of labor [2,4,9,12,13,14,15]. At the electrophysiological level, UCs are initiated by the synchronized electrical activity of excitable myometrial cells, which can be recorded from the maternal abdominal surface [16,17]. By recording uterine bioelectrical activity, EHG provides electrophysiological information distinct from the mechanical measures obtained using conventional uterine monitoring methods [2,5,11,17]. Compared with conventional monitoring approaches, EHG has shown favorable performance in terms of sensitivity and diagnostic accuracy in detecting contractions and characterizing labor [3,4,13,17,18,19,20,21]. These advantages may provide deeper insights into labor progression and the effectiveness of UCs beyond simple contraction detection.

Despite these advantages, the clinical adoption of EHG remains limited due to fragmentation across electrode design, electrode topology, wearable implementation, signal processing, and clinical validation. A significant challenge identified in the literature is the absence of standardized electrode topologies and integrated design frameworks [18]. The current EHG literature reflects highly heterogeneous electrode configurations, ranging from sparse bipolar channels to dense arrays, resulting in inconsistent spatial sampling and limited reproducibility. Additionally, substantial variability in signal processing methodologies hampers the comparability of findings across studies and constrains the development of standardized clinical workflows. Furthermore, although high-density grids provide greater spatial sampling and facilitate detailed propagation mapping, they often compromise clinical practicality by increasing setup complexity and reducing long-term patient comfort [12]. Conversely, low-density electrode topologies enhance deployment feasibility but often compromise signal fidelity and spatial resolution. Achieving a balance between reliable signal acquisition and practical usability remains a significant challenge. Factors such as patient comfort, user acceptance, and compatibility with clinical workflows are critical for the widespread adoption of continuous maternal monitoring. However, issues including prolonged skin contact, inconsistent electrode adhesion, and restricted mobility continue to impede practical usability [11,21]. Recent advances in wearable and wireless technologies aim to address these challenges by facilitating ambulatory monitoring and improving user comfort [22,23]. Despite these advances, a substantial gap persists between experimental developments in EHG and clinically standardized solutions suitable for routine labor monitoring [4,11,24].

To address this gap, this study critically synthesizes EHG-based monitoring systems, focusing on electrode placement and configuration, signal acquisition methodologies, wearable implementation, and clinical applicability. Particular emphasis is placed on the effects of electrode placement, motion artifacts, signal processing, wearability, and clinical integration on the assessment of UA and labor progression. Previous reviews have examined important aspects of EHG monitoring, including electrode count, placement, supporting electrodes, signal characteristics, and clinical applications [15,18,25]. Building on these contributions, the present review broadens the scope by integrating evidence on electrode configuration, wearable implementation, and clinical feasibility, while distinguishing direct EHG evidence from transferable technical evidence derived from related biopotential-monitoring technologies. This integrated perspective enables the relationships among acquisition approaches, wearable implementation constraints, signal reliability, usability, and clinical feasibility to be considered within a common framework. Rather than advocating for a specific system, this review consolidates the literature to identify factors influencing the reliability and clinical utility of wearable EHG monitoring. By synthesizing current findings, it aims to identify key limitations, emerging trends, and future directions for the development of reliable and clinically viable wearable EHG systems.

## 2. Background

### 2.1. Limitations of Current EHG Systems: A System-Level Perspective

High-density EHG studies demonstrate that uterine electrical activity propagates as discrete spikes with quantifiable velocity and directional characteristics, analogous to myocardial conduction [16,17]. However, the reliability of these measurements is highly dependent on electrode configuration, spatial coverage, and inter-electrode spacing. In practice, large inter-electrode distances (>3 cm) and heterogeneous electrode layouts often result in inconsistent propagation mapping and limited comparability across studies [16]. Sparse or bipolar configurations further constrain spatial information and remain highly sensitive to electrode orientation relative to the uterine pacemaker [16,17]. Collectively, these limitations highlight the lack of a standardized spatial sampling framework, thereby reducing reproducibility and clinical reliability.

This challenge extends to acquisition methodologies, for which consensus on electrode count, placement strategy, and recording protocol remains lacking [18,26]. Although EHG sensitivity remains stable in obese patients [8], commonly employed electrode topologies, such as configurations using four abdominal electrodes with reference and ground electrodes [27], are largely empirical and have not been systematically optimized. Furthermore, signal acquisition is often influenced by physiological and environmental factors, including maternal movement and external disturbances, which can compromise signal robustness and increase measurement variability [7]. Consequently, EHG recordings remain highly dependent on the experimental setup, limiting cross-study validation and hindering clinical translation.

From a monitoring-performance perspective, EHG appears to be less affected by maternal obesity than conventional monitoring methods [15,28,29]. In obese women, EHG contraction-detection sensitivity has been reported to range from 82.0% to 90.2%, compared with 45.8% to 65.3% for TOCO; in morbidly obese women (BMI >40 kg/m^2^), the median sensitivity was 87.2% for EHG versus 45.0% for TOCO [15]. Although increasing BMI and abdominal circumference may affect monitoring performance, their reported associations with EHG performance were generally not statistically significant [15]. Furthermore, clinical feedback indicates favorable usability in specific contexts [8]. However, these advantages are limited by the inherent constraints of electrode interfaces. Although conventional Ag/AgCl patch electrodes are widely used for clinical biopotential monitoring, their performance during extended monitoring is often compromised by gel dehydration and adhesive degradation. These issues can result in skin irritation and signal loss [30]. Furthermore, these systems have a limited ability to resolve the spatiotemporal directionality of uterine electrical signals [31]. Increased subcutaneous tissue thickness further attenuates high-frequency components, complicating feature extraction [28,29].

To improve comfort and flexibility, wearable and textile-based electrodes have been developed. However, these solutions introduce significant challenges in maintaining signal stability. Maternal movement and abdominal deformation alter contact impedance, generating non-stationary noise that disrupts advanced signal processing techniques [32,33,34]. Moreover, the absence of mechanical stabilization complicates consistent electrode placement, undermining the reliability of connectivity and clustering analyses [24,35,36]. Recent advances in flexible and stretchable electronic systems, including printed circuit board (PCB)-based wearable arrays, offer potential solutions to these challenges, but they remain largely experimental and lack comprehensive clinical validation [37]. Therefore, wearable designs must prioritize both signal fidelity and user comfort to achieve practical usability.

Overall, continuous uterine monitoring remains challenging because conventional methods often require transducer repositioning, while EHG acquisition may require careful skin preparation and subsequent electrode removal [7]. A previous study [15] reported positive predictive values ranging from 78.7% to 92.0% for EHG-based contraction detection, further supporting its potential for uterine activity monitoring. However, EHG signal reliability continues to be influenced by electrode configuration, electrode–skin impedance, acquisition conditions, and patient-specific variability [15,38]. Current EHG systems are constrained by four interrelated limitations:

(1) Lack of standardized electrode topology.
(2) Instability of the electrode–skin interface.
(3) Variability in acquisition and implementation approaches.
(4) Dependence of signal reliability on electrode placement and patient-specific variability.

### 2.2. Comparative Analysis of Existing EHG Systems and Transferable Biopotential-Monitoring Technologies

EHG systems can be categorized into (i) wired clinical systems, (ii) patch-based systems, and (iii) wearable or textile-integrated systems [6,14,39,40,41,42,43,44,45,46,47,48,49,50,51]. These categories differ in signal quality, practical usability, and clinical applicability. Each category addresses specific limitations of conventional UA monitoring while introducing new technical and usability challenges [11,21,22]. Wired electrode configurations typically provide reliable signal acquisition but restrict mobility and long-term usability (Table 1).

Wearable EHG development draws primarily on evidence from systems designed to acquire uterine bioelectrical activity. However, several technical components relevant to wearable EHG, including textile and dry electrodes, wearable electrode configurations, electrode–skin interfaces, and reference-signal acquisition, have also been extensively investigated in ECG, EMG, fetal ECG, and other biopotential-monitoring applications. These signals are typically acquired using systems such as noninvasive fetal electrocardiography and integrated platforms such as the Nemo fetal monitoring system, in which electrode placement is critical for supporting usable signal acquisition and interpretability. Standard electrode topologies typically use abdominal electrode placement (P1) to simultaneously capture both cardiac and uterine signals, with reference and ground electrodes positioned near the lower abdomen (P2) [39]. Alternative electrode configurations (P3) involve electrode placement at different body locations, including the upper right and left shoulders, the left side beneath the abdomen [43], as well as other locations on the body [45], the upper body [46,47,49], and the forearm [51]. These alternative topologies have been investigated for signal discrimination and multimodal monitoring. The diversity in electrode placement strategies highlights the absence of a universally accepted standard for EHG electrode configuration, as usable signal acquisition is highly dependent on precise spatial alignment with UA. Consequently, electrode topology remains an important design consideration that directly affects the reliability of biopotential-monitoring applications.

Accordingly, this section distinguishes evidence obtained directly from EHG studies from transferable technical evidence derived from related biopotential-monitoring technologies. The latter is considered only in relation to technical implementation relevant to wearable EHG and is not interpreted as direct evidence of EHG-specific clinical performance (Table 1). Configurations range from low-density topologies (2–3 electrodes) to multichannel arrays (up to 12 electrodes). Most hospital-based implementations favor abdominal (P1) placements for signal acquisition during intrapartum monitoring [5,14,39,40,41,42,43,44]. By contrast, alternative electrode configurations such as P2 and P3 are more frequently investigated in ambulatory or research settings, where flexibility and multisignal acquisition are prioritized [39,43,45,46,47,49,51]. Despite these developments, no consensus has been reached regarding a practical electrode topology, and usable signal acquisition varies considerably across systems and clinical scenarios.

Electrode placement strategies remain application-dependent, with substantial variation in electrode count, abdominal placement, and spatial distribution across studies. Increasing electrode coverage may improve spatial sampling but can also increase implementation complexity, whereas simplified configurations may facilitate practical use at the expense of spatial information. This variability reflects the absence of a unified framework that effectively links electrode configuration to clinical performance outcomes. Patch-based systems typically employ distributed adhesive electrode arrangements to enhance portability and facilitate deployment in both clinical and research settings [5,14,39,40,41,42,43,44]. Although these systems are often reported to be well tolerated and operationally practical [39,42,44,46,50], their performance may be affected by motion artifacts, electrode displacement, fetal movement, tissue characteristics, and other sources of physiological variability [5,14,39,41,42,44]. As summarized in Table 2, these factors can contribute to signal degradation, noise susceptibility, and reduced robustness under real-world monitoring conditions [14,39,42,45,47]. Although these systems offer practical advantages, their robustness under real-world monitoring scenarios remains limited. Wearable and textile-based systems represent a recent advancement in EHG monitoring, emphasizing continuous and ambulatory data acquisition through the integration of conductive materials into garments [45,47,48,49,50]. These systems may enhance comfort, mobility, and long-term wearability, thereby supporting potential home care and telemonitoring applications [40,44,47,49,50]. Studies of related wearable biopotential-monitoring technologies suggest that textile electrodes may support prolonged skin contact and wearable integration, although EHG-specific validation remains necessary [14,42,45,46,47,48,50,51]. In Table 2, the terms “comfortable” and “well tolerated” are retained as review-level categories synthesized from qualitative usability descriptions in the cited studies rather than from standardized quantitative measures. Additional challenges include baseline drift, placement-dependent variability, and reduced reproducibility during ambulatory monitoring.

Clinically feasible EHG systems must integrate electrode topology, hardware architecture, and usability within a unified framework. Current solutions tend to prioritize either signal quality (wired systems) or usability (wearable systems), with patch-based systems representing a compromise between these priorities, without fully addressing the inherent trade-offs. Furthermore, the absence of a unified comparative framework in previous studies has contributed to fragmentation and limited the systematic evaluation of performance across different system designs. Inconsistencies in electrode topology, evaluation metrics, and experimental conditions further hinder standardized comparisons and delay the clinical translation of EHG monitoring technologies.

### 2.3. Design Requirements for Clinically Deployable Wearable EHG Systems

The limitations outlined in Section 2.1 emphasize the importance of integrated design strategies that effectively balance signal fidelity, usability, and clinical feasibility. Future EHG systems should adopt a holistic approach that coordinates electrode design, acquisition stability, and signal processing, rather than optimizing each component in isolation [18]. A critical requirement is the reliable differentiation between Braxton–Hicks contractions and active labor [28], which remains a challenge for current monitoring technologies. Advanced signal processing techniques, including wavelet transforms and entropy-based methods, have shown promise in detecting electrophysiological transitions indicative of labor onset [9,23]. However, as highlighted in Section 2.1, the efficacy of these methods is highly dependent on signal quality, emphasizing the need for robust acquisition systems. Although high-density surface electrode arrays support more detailed spatial resolution [52], existing solutions often lack flexible, textile-integrated designs capable of maintaining signal integrity during maternal movement and postural changes [44,48,53,54,55].

A structured questionnaire incorporating continuous and ordinal rating scales was used to assess clinicians’ experiences with EHG, including knowledge, competence, usability, skin preparation, impedance checks, technical issues, and skin irritation. The results indicate that future EHG systems should be wireless and waterproof, and incorporate integrated impedance monitoring to enhance mobility and user satisfaction [7]. These advancements could also facilitate home-based monitoring in remote areas [56]. Furthermore, EHG may provide additional electrophysiological information compared with conventional monitoring methods [15], while noninvasive approaches are generally preferred because of their safety and greater clinical acceptance [8]. From a hardware and materials perspective, the development of wearable EHG systems must carefully consider long-term comfort, skin compatibility, and mechanical stability. The use of conductive gels and adhesive electrodes should be minimized or avoided, as these materials are associated with skin irritation and discomfort during extended monitoring periods [45,47,50]. Textile-based electrode technologies, such as conductive fabrics incorporating nanofibers coated with conductive polymers, represent a promising alternative by enabling stable biopotential acquisition while maintaining excellent skin compatibility [51]. However, as discussed in Section 2.1, wearable systems are susceptible to motion artifacts and contact instability. Therefore, electrode placement must be mechanically stabilized within the wearable structure to ensure consistent positioning throughout monitoring. Elastic wearable designs, including abdominal belts or fabric wraps, can improve conformity to body contours and enhance signal stability [17,46,48,51].

Additional practical considerations include device maintenance and hygiene. Accordingly, wearable monitoring garments should be constructed from washable materials to facilitate repeated clinical use without compromising electrical performance [45,47,50]. Furthermore, modular electrode designs that allow repositioning or removal are advantageous because they provide flexibility in accommodating variations in abdominal anatomy and diverse clinical measurement requirements [51].

## 3. Methodology

**Figure 1:**
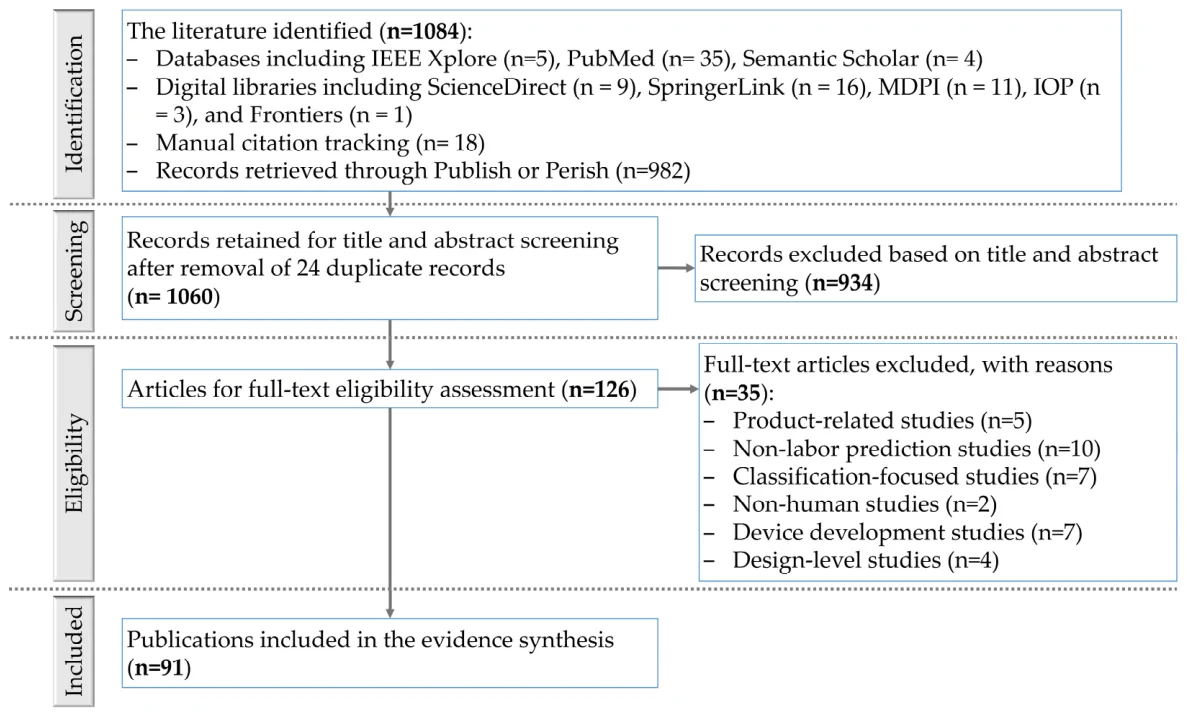
PRISMA flow diagram illustrating record identification, screening, eligibility assessment, and inclusion in the systematic review.

A systematic literature review was conducted following PRISMA 2020 principles (Figure 1), with the study selection process documenting the identification, screening, eligibility assessment, and inclusion of relevant studies. A completed PRISMA 2020 checklist is provided in Supplementary Table S3 to document the corresponding reporting items and their locations in the manuscript. No formal systematic review protocol was prepared or prospectively registered before the review was conducted. Title and abstract screening was initially performed by the first author according to the predefined eligibility criteria. Full-text eligibility assessment was also initially conducted by the first author. The screening and eligibility decisions were subsequently checked by a co-author with expertise in engineering and then reviewed by another co-author with expertise in the medical aspects relevant to the review. Any disagreements or uncertainties regarding study eligibility were resolved through discussion among the authors until consensus was reached. No formal methodological quality or risk-of-bias assessment was performed. Study inclusion was based on the predefined eligibility criteria and relevance to the review objectives rather than on a formal quality-rating threshold. The studies included in the systematic review comprise two evidence categories: direct EHG evidence and transferable technical evidence. Direct EHG evidence includes studies specifically investigating EHG or uterine bioelectrical monitoring, whereas transferable technical evidence includes selected studies of related biopotential-monitoring technologies, such as ECG, EMG, and fetal ECG, that provide technical findings relevant to electrode materials and configurations, wearable integration, electrode–skin interfaces, reference-signal acquisition, or artifact management. Transferable technical evidence is considered only for aspects relevant to wearable EHG implementation, and modality-specific diagnostic or clinical findings are not extrapolated to EHG.

To maintain methodological rigor, search targets were organized into two categories: indexing databases (including IEEE Xplore, PubMed, and Semantic Scholar) and publisher-specific digital libraries (specifically ScienceDirect, SpringerLink, MDPI, IOP, and Frontiers). Additional records were identified through manual citation tracking and searches conducted using Publish or Perish (version 8) software, which was also used to query Crossref. The search was conducted on 9 January 2026 and was configured to retrieve up to 1000 records. The initial search yielded 1000 records, of which 18 incomplete records were removed before consolidation with the other search results, leaving 982 records from the Publish or Perish search. These records were subsequently consolidated with those obtained from the other information sources and subjected to the same deduplication and screening procedure. The search included studies published from September 1997 to January 2026. The development of EHG research showed rapid growth from 2022 to 2026, particularly in the areas of artificial intelligence and signal processing, following a transitional period (2017–2021) that focused on wearable systems and earlier foundational developments before 2016. Literature identification was conducted between 6 October 2025 and 12 March 2026 across the information sources specified above. Except for the Publish or Perish/Crossref search conducted on 9 January 2026, exact source-specific search dates were not retained. Therefore, the overall literature-identification period is reported rather than retrospectively reconstructed source-specific dates. The searches were based on combinations of terms relating to EHG, wearable monitoring, electrodes or sensors, and obstetric applications, with syntax adapted to the search functionality of each information source. A structured search strategy was implemented using combinations of keywords and Boolean operators. The primary search string was formulated as follows: (“electrohysterography” OR “EHG”) AND (“wearable” OR “garment”) AND (“electrode” OR “sensor” OR “topology”) AND (“labor” OR “uterine contraction” OR “pregnancy”). The search strategy was adapted to each information source while maintaining the core concepts of EHG, wearable or electrode-based monitoring, and pregnancy- or labor-related applications. The database-, digital-library-, and manual-tracking search queries are provided in Supplementary Table S1, while the studies included in the systematic review and their evidence classifications are summarized in Supplementary Table S2 to enhance transparency and reproducibility.

To ensure methodological rigor, studies were selected according to clearly defined inclusion and exclusion criteria centered on EHG systems. The search terms reflected the review’s focus on wearable EHG acquisition and electrode implementation. Nevertheless, requiring combinations of EHG, wearable-related, electrode-related, and obstetric terms may have reduced search sensitivity because some relevant studies may use different terminology or may not explicitly describe the monitoring approach as wearable. The search was conducted across multiple indexing databases and publisher-specific digital libraries and was supplemented by Publish or Perish searches and manual citation tracking to identify additional relevant literature. However, these approaches do not eliminate the possibility that relevant studies were missed.

Publications were eligible if they provided evidence relevant to the development, implementation, or evaluation of wearable EHG monitoring systems, including electrode topology, electrode placement, electrode–skin interfaces, reference-signal acquisition, and wearable system integration. Primary experimental studies were included as direct evidence, while selected review articles were retained when they provided directly relevant secondary technical syntheses. Review articles were not treated as independent primary-study observations in quantitative summaries. Studies were excluded if they did not provide sufficient technical information relevant to the scope of the review, focused exclusively on topics unrelated to wearable EHG implementation, lacked experimental or technical evidence applicable to the review objectives, involved non-human subjects, or were unavailable in full-text. For PRISMA reporting, full-text exclusions were assigned to a single primary exclusion category. Product-related, classification-focused, device-development, and design-level reports were excluded if they did not provide technical evidence applicable to wearable EHG implementation. Non-labor-prediction studies were excluded when their clinical objective fell outside the scope of this review, while non-human studies were excluded according to the predefined population criterion (Figure 1). This selection process retained publications that were relevant to the review objectives and provided sufficient technical detail for the evidence synthesis.

The literature search identified a total of 1084 records (Figure 1). These included 44 records from indexing databases (IEEE Xplore, *n* = 5; PubMed, *n* = 35; Semantic Scholar, *n* = 4), 40 records from publisher-specific digital libraries (ScienceDirect, *n* = 9; SpringerLink, *n* = 16; MDPI, *n* = 11; IOP, *n* = 3; Frontiers, *n* = 1), 18 records identified through manual citation tracking, and 982 records retrieved through Publish or Perish. The records retrieved through Publish or Perish (*n* = 982) were imported into the reference management software and stored in a separate folder from those obtained from the other information sources. After all retrieved records were consolidated in Zotero, duplicate records were identified using Zotero’s “Duplicate Items” function. After removal of 24 duplicate records, 1060 unique records were retained for title and abstract screening. Of these, 934 records were excluded, leaving 126 articles for full-text eligibility assessment. Following full-text assessment, 35 articles were excluded, resulting in 91 publications being included in the evidence synthesis. In addition to the included studies, four standards and guidance documents were cited separately to provide relevant technical, safety, and regulatory context. These documents were not treated as eligible studies and were therefore excluded from the PRISMA study-selection count. Accordingly, the manuscript contains 95 references in total, comprising 91 publications included in the evidence synthesis and four additional standards or guidance documents used for contextual purposes. An included study was not required to report every technical parameter examined in this review. Therefore, “Not reported” in the summary tables indicates that the study met the eligibility criteria but did not explicitly report the specific parameter being summarized. It does not indicate that the study provided insufficient technical information for inclusion.

## 4. Results

For descriptive comparison across the reviewed studies, electrode configurations were grouped into three density ranges based on the distribution of electrode counts observed in the included literature [12,52,57,58,59]. This categorization was developed by the authors for the purposes of this review and was not derived from an established standard or validated electrode-density classification framework. The ranges are therefore intended to facilitate comparison of reported EHG configurations rather than to define recommended or optimal electrode densities. Multi-electrode configurations range from medium-density arrays with eight electrodes to high-density grids with 16 electrodes. These configurations support detailed characterization of uterine electrical propagation, synchronization, and connectivity. However, the advantages of increased electrode density are often offset by greater system complexity and longer setup times, leading to a trend in the literature toward practical trade-offs rather than maximizing electrode density. To mitigate signal contamination from power line interference and maternal or fetal cardiac activity, advanced processing techniques such as independent component analysis (ICA), zero-crossing rate analysis, and methods leveraging slow–fast wave interactions are typically employed [17,38,60,61]. Concurrently, recent advancements in flexible, screen-printed, and textile-based electrodes have facilitated wearable solutions [27,47,51,62,63], enabling seamless integration into adjustable maternity garments or patches to enhance electrode–skin contact. Long-term stability, motion-induced variability, and consistency of signal acquisition remain major challenges for widespread clinical deployment [30].

### 4.1. Electrode Topology: Quantitative Trade-Offs and Design Implications

#### 4.1.1. Electrode Density and Spatial Resolution

**Figure 2:**
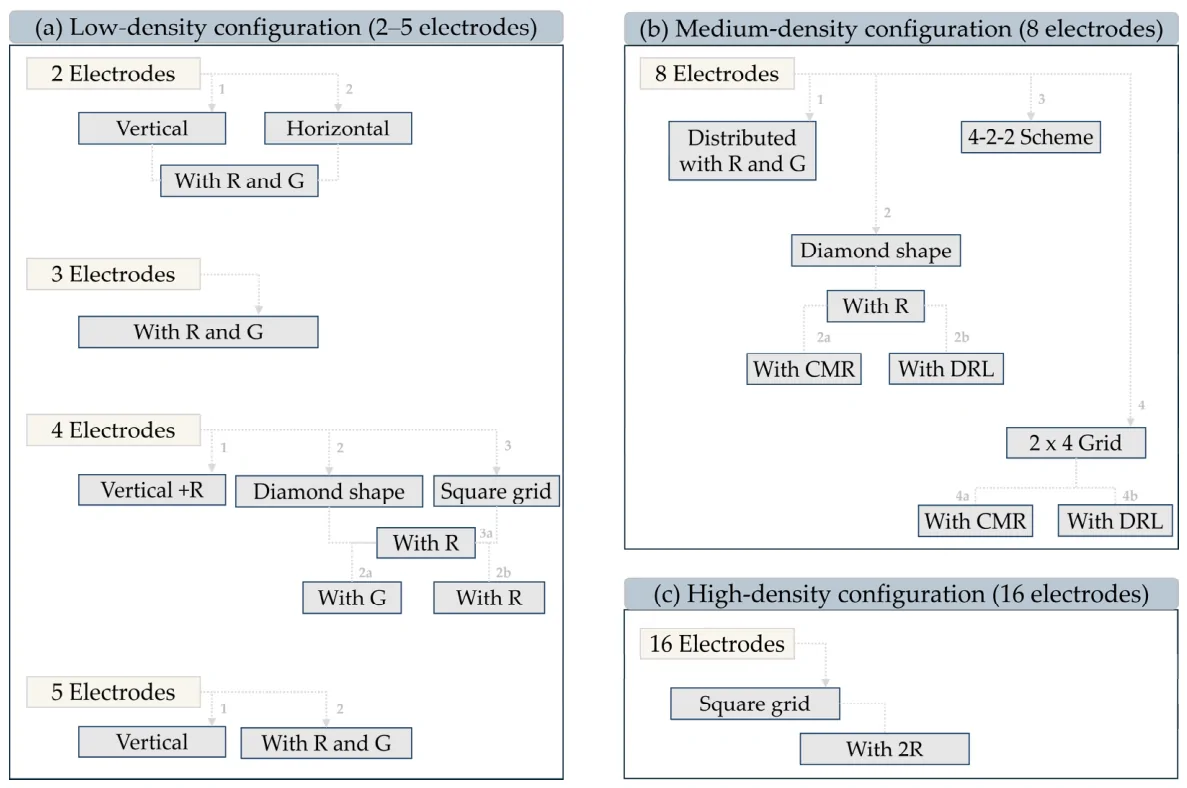
Classification of electrode topologies according to electrode density and spatial arrangement. (**a**) Low-density topologies with two to five electrodes, including vertical, horizontal, and diamond placements. (**b**) Medium-density topologies with eight electrodes arranged in distributed, diamond, 4–2–2, or 2 × 4 grid layouts. (**c**) High-density topologies comprising a 16-electrode square grid array with two reference electrodes and a typical inter-electrode spacing of 17.5–25 mm. Numbers and letter suffixes (e.g., 1, 2a, and 2b) indicate configuration variants and subvariants within each electrode-count group; R, G, CMR, and DRL denote reference, ground, common-mode reference, and driven-right-leg electrodes, respectively [3,12,18,57,58,61,64,65,66,67,68,69,70].

The acquisition of high-fidelity EHG signals is critically influenced by electrode topology, encompassing factors such as electrode count, electrode arrangement, inter-electrode spacing, and the repositioning of the reference electrode. Given the complex, multidirectional propagation of uterine electrical activity, electrode density is crucial for capturing synchronized myometrial bursts and delineating the extent of propagation coverage [12,57,58,59]. For descriptive comparison across the reviewed studies, electrode configurations were grouped into three density ranges, namely low-density (2–5 electrodes), medium-density (8 electrodes), and high-density (16 electrodes) arrays (Figure 2), based on the configurations reported in the included literature [1,2,3,4,9,12,13,19,21,23,24,26,30,31,32,36,38,40,42,56,57,58,59,61,64,65,66,67,68,69,70,71,72,73,74,75,76,77].

This categorization was developed by the authors for the purposes of this review and is not derived from an established standard or validated electrode–density classification framework. Among the 50 primary studies classified as direct EHG evidence, 18 (36.0%) reported electrode counts within the low-density range of 2–5 electrodes, 5 (10.0%) used 8-electrode configurations, and 15 (30.0%) used 16-electrode configurations. Five studies (10.0%) reported other electrode counts, 1 (2.0%) reported a range spanning multiple density groups, and 6 (12.0%) did not provide sufficient information for unambiguous classification (Table S2). The ranges are therefore intended to facilitate comparison of reported EHG configurations rather than to define recommended or optimal electrode densities.

Low-density topologies, often arranged in simple vertical, horizontal, diamond, or basic grid patterns with reference, ground, or driven-right-leg (DRL) electrodes, are favored for their clinical practicality. However, their limited propagation detail constrains the analysis of propagation patterns and diminishes the reliability of UA mapping. Medium-density arrays, typically comprising 8 electrodes in structured layouts such as 4–2–2 or 2 × 4 configurations, enhance spatial coverage and enable multichannel characterization of propagation and synchronization while maintaining moderate system complexity. High-density grids with 16 electrodes provide the most comprehensive spatial information, facilitating advanced analyses of uterine propagation, connectivity, and synchronization. These advantages, however, are offset by increased setup complexity, higher acquisition demands, and reduced patient comfort during extended monitoring periods [3,12,18,57,58,61,64,65,66,67,68,69,70].

**Figure 3:**
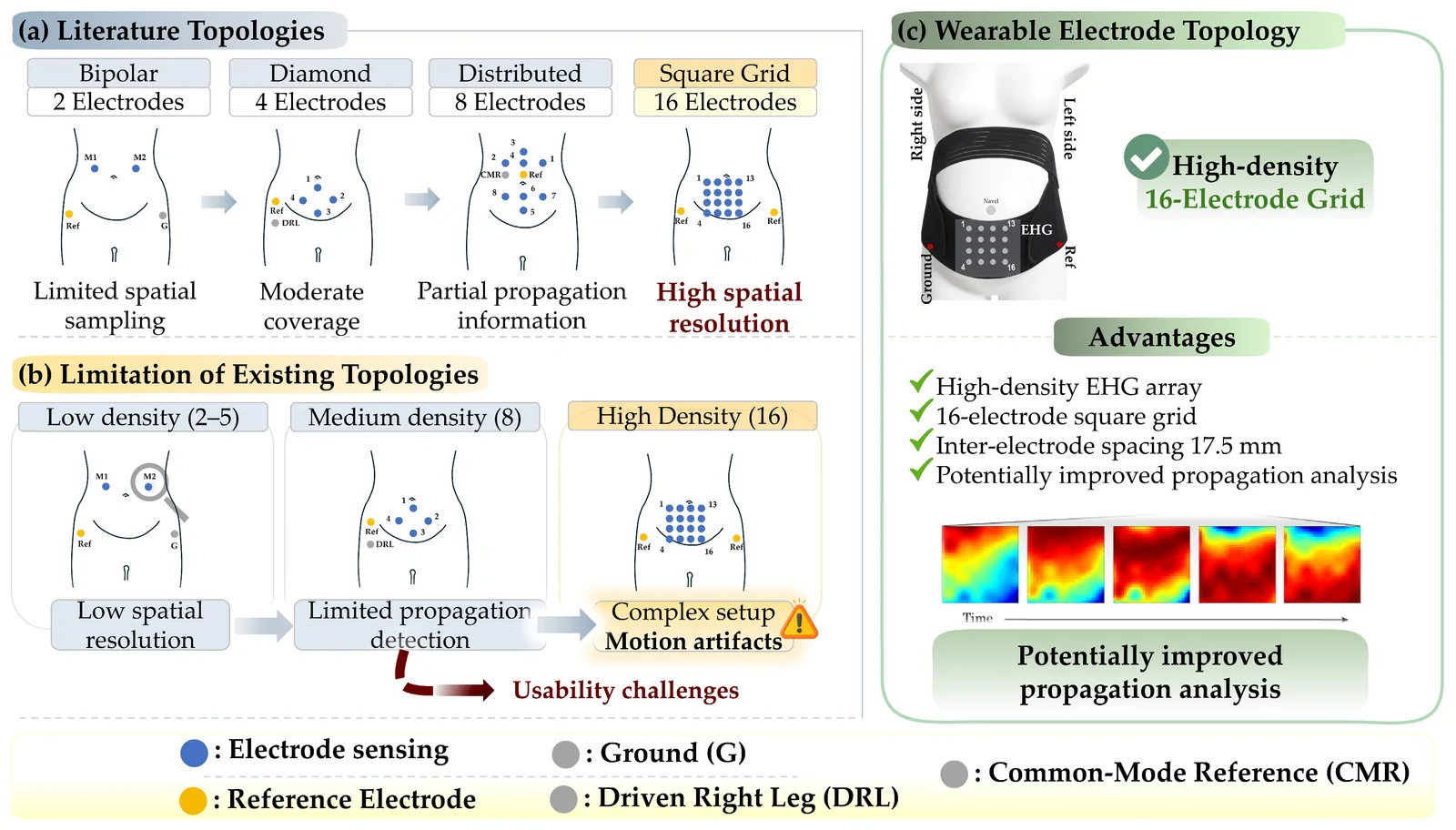
Comparative interpretation of EHG electrode topologies: (**a**) Electrode layouts reported in the literature ranging from bipolar recordings to distributed arrays. (**b**) Key limitations of existing topologies, including low spatial resolution, limited propagation detection, and incomplete spatial coverage. (**c**) High-density wearable electrode topologies as a commonly reported practical approach, including 4 × 4 grid layouts positioned below the navel, with reference electrodes placed at a distance (e.g., >20 cm) to support spatial mapping of uterine electrical activity. Letter labels (e.g., M1, M2) and numbers denote individual electrode positions and counts as reported in the literature. Author-developed schematic synthesized from [3,12,18,57,58,61,64,65,66,67,68,69,70].

Taken together, the reviewed studies indicate that configurations employing approximately 8–16 electrodes represent a frequently reported practical range for achieving sufficient spatial sampling while balancing spatial characterization and implementation complexity. This range should be interpreted as a literature-derived benchmark rather than a universally validated optimum, because the appropriate electrode count depends on the monitoring objective, required spatial information, electrode arrangement, and clinical setting [18,20,65]. Inter-electrode spacing influences the spatial sampling of uterine electrical activity and the characteristics of the differential EHG signal, but does not directly determine the common-mode rejection ratio (CMRR). CMRR is primarily a characteristic of the differential acquisition front end and can also be affected by an imbalance in the electrode–skin and input impedances [42,43]. Electrode configurations have evolved from simple bipolar arrangements to high-density arrays, supporting the analysis of signal propagation (Figure 3).

These design considerations directly influence the efficacy of advanced signal processing techniques. For example, ICA can exploit multichannel spatial diversity to separate UA from maternal ECG and other interference, while zero-crossing rate analysis provides complementary temporal information [17,60,61,78]. Furthermore, electrode topology must remain robust to changes in maternal posture, such as semi-Fowler’s position or lateral tilts, as these variations can significantly impact the electrode–skin interface and physiological signal characteristics [53,79]. To address this variability, standardized topologies informed by computational dipole-band models have been developed to enhance the consistency and robustness of signal acquisition across diverse obstetric scenarios [20]. These strategies facilitate applications such as cervical dilation assessment and preterm labor prediction [1,71,72], although their effectiveness remains contingent upon stable electrode placement and signal quality. Consequently, reliable obstetric monitoring demands not only high spatial resolution but also a robust system design that can adapt to both clinical and ambulatory environments [18,22,73].

EHG electrode placement is typically described using topology-based layouts that specify the arrangement of sensing electrodes on the maternal abdomen. As outlined in Table 3, the principal topologies include vertical (V), horizontal (H), diamond (D), square-grid (SG), distributed (DI), scheme-based (SS), and rectangular-grid (RG) configurations. These abbreviations are consistently used throughout the manuscript to refer to the respective electrode topologies.

Table 3 further differentiates the types of supporting electrodes associated with each topology, including reference (R), ground (G), combined reference–ground (R&G), DRL, and common-mode reference (CMR). In biopotential acquisition systems such as ECG, EEG, and EMG, DRL and CMR configurations are employed to minimize common-mode interference—particularly 50/60 Hz power-line noise—thereby enhancing signal stability. This distinction is important because electrode configuration determines the spatial coverage, whereas supporting electrodes primarily influence noise suppression and baseline stability. Table 3 summarizes the trade-offs among spatial coverage, implementation complexity, and practical usability across the reported electrode configurations, highlighting the need for application-specific electrode selection rather than a universally optimal configuration.

#### 4.1.2. Design Justification of Electrode Topology Based on Uterine Electrical Propagation Dynamics

The transition from quiescent UA to active labor is characterized by increased electrical coupling among myometrial cells, resulting in accelerated propagation velocity (PV) of uterine signals. This physiological shift serves as a robust biomarker for differentiating true labor from Braxton–Hicks contractions [19]. In one study of symptomatic patients, a predictive model integrating propagation velocity with frequency-domain markers achieved an area under the receiver operating characteristic curve (AUROC) of 0.96 and 100% sensitivity for identifying imminent delivery [2]. These findings are study-specific and require validation in broader and independent populations before being generalized to clinical EHG performance. Nevertheless, they suggest that characterization of propagation dynamics may provide clinically relevant information and support its consideration in EHG acquisition and analysis.

Previous studies indicate that abdominal EHG can be affected by physiological interference from non-uterine sources, which can complicate the interpretation of uterine electrical activity [1]. Multichannel EHG recordings can also characterize the spatial distribution and propagation of uterine electrical activity. High-resolution EHG studies reveal that uterine electrical bursts follow spatiotemporal patterns closely mirroring those of mechanical pressure waves, with bidirectional propagation across the uterus [16]. Spatial mapping techniques, combined with Gaussian fitting applied to electrode grid data, enable precise localization of the UA center and comprehensive assessment of myometrial functional connectivity [14,16]. However, these advanced analyses necessitate sufficiently dense and well-distributed electrode arrays, as limited spatial sampling can hinder accurate reconstruction of propagation pathways. For example, a 16-electrode two-dimensional grid has been used to estimate both the velocity and direction of EHG propagation, with no single preferred propagation direction identified. Such propagation information may provide additional value for the clinical assessment of uterine activity when evaluation extends beyond contraction detection; however, this does not establish that 16 electrodes are clinically necessary for routine monitoring [19].

Both electrode density and inter-electrode spacing are critical determinants of the ability of the system to capture uterine propagation features (see Table 4). Low-density topologies provide more limited spatial information for reliable estimation of PV and direction, whereas high-density arrays support spatial resolution but introduce motion sensitivity and reduced usability. Furthermore, inter-electrode spacing directly influences signal independence: excessive spacing can result in undersampling, whereas overly narrow spacing may lead to signal redundancy and increased crosstalk. The available evidence does not establish a validated minimum electrode count or inter-electrode spacing for propagation-velocity or graph-based connectivity analyses; reported requirements remain algorithm- and application-dependent.

### 4.2. Wearable System Design: From Literature to Design Guidelines

Wearable EHG systems must carefully balance patient safety, signal reliability, and clinical usability. Recent research increasingly highlights the importance of standards-compliant, ergonomically adaptive, and reliable wearable designs to facilitate clinical adoption. From a safety-assessment perspective, adherence to relevant standards such as IEC 60601-1 [80] can support the evaluation of basic safety and essential performance for continuous abdominal biopotential monitoring [15,42]. Recommended safety measures include electrical isolation, controlled leakage currents, and the incorporation of fail-safe protection mechanisms [80,81]. Additionally, the use of biocompatible electrode materials is essential to support stable electrode–skin impedance and minimize irritation during extended wear [82]. However, validation of long-term safety under real-world clinical conditions remains limited.

From a design perspective, flexible and textile-based electrode systems have emerged as a leading solution, offering improved conformity to abdominal contours and enhanced signal consistency compared with rigid or adhesive alternatives [55,62,74]. However, these innovations introduce greater design complexity and increased susceptibility to motion artifacts, reflecting persistent compromises between signal quality and usability. Integrated wearable solutions have been investigated as an alternative to conventional patch-based systems, with individual studies reporting potential advantages in setup efficiency and patient acceptance [25,37]. Their performance nevertheless remains dependent on factors including electrode placement and patient-specific anatomical variability [7,56]. Ultimately, current wearable EHG designs remain constrained by the need to balance safety, mechanical stability, and signal robustness.

#### 4.2.1. Wearable Design Considerations

Current evidence demonstrates that wearable EHG systems must ensure stable electrode–skin contact, adaptability to maternal posture, and sustained user comfort to achieve reliable signal acquisition. Flexible and textile-based electrodes offer conformity to abdominal contours and reduce discomfort compared with rigid or adhesive alternatives [55,62,74]. Maternal posture (e.g., supine tilt, lateral, and semi-recumbent) influences both signal characteristics and user experience [53,83]. However, current designs demonstrate limited robustness to posture-induced electrode displacement. Integrated belt-based systems enhance placement repeatability and workflow efficiency [7,56]. Conversely, their performance remains contingent on user-specific anatomical variability and consistent positioning, highlighting the absence of standardized placement protocols across studies. Consequently, signal quality often varies substantially, particularly in real-world clinical settings.

Current wearable designs are often developed without fully integrating considerations of electrode topology and signal processing requirements. Systems with suboptimal stabilization or spatial topology frequently rely on extensive post-processing compensation, which diminishes robustness and generalizability. To date, wearable EHG systems have not fully resolved the trade-off between comfort, stability, and signal fidelity.

#### 4.2.2. System-Level Architecture of Wearable EHG Systems

**Figure 4:**
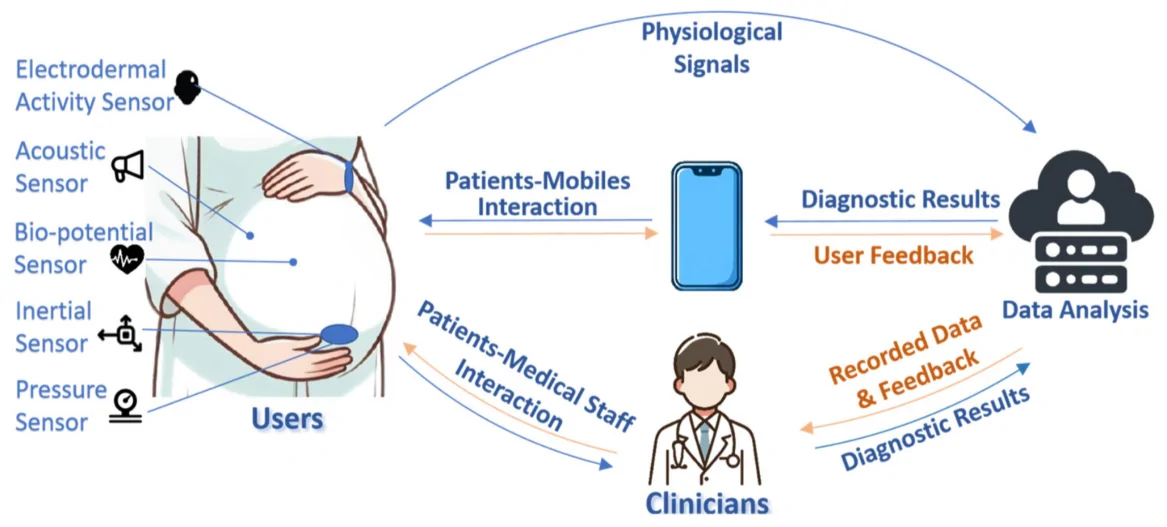
Wearable maternal monitoring ecosystem integrating physiological sensors, mobile interface, clinician interaction, and data analytics. Reproduced from Liu et al. [84] under the Creative Commons Attribution 4.0 International (CC BY 4.0) license.

Wearable EHG systems are typically described as having a layered architecture comprising acquisition, processing, and output components [78,81,82,83]. A modular maternal monitoring ecosystem comprising physiological sensing, embedded processing, and clinical interface components is shown in Figure 4 (reproduced from [84]). While this abstraction facilitates scalability and interoperability, existing implementations remain predominantly component-driven, with limited integration across layers. At the acquisition level, wearable systems utilize surface electrodes embedded within flexible substrates to capture uterine biopotentials [48]. However, as detailed in Section 2 and Section 4.1, the fidelity of these signals is critically influenced by electrode topology, spatial distribution, and motion robustness. As shown in Figure 4, the variability introduced at this stage propagates through subsequent layers, directly impacting overall system performance. This indicates that acquisition quality is not an isolated consideration. Rather, it fundamentally constrains all downstream processing and interpretation. The literature consistently highlights that reliable signal acquisition requires low-noise amplification, appropriate filtering, and robust differential signal conditioning, particularly under challenging conditions such as maternal movement or obesity [4,29]. These findings primarily characterize the technical reliability of EHG acquisition and should not, in isolation, be interpreted as evidence of clinical feasibility. Multimodal topologies that integrate fetal ECG and EHG can provide richer physiological insights but also introduce additional challenges related to synchronization and artifact management [5]. Overall, the evidence indicates that acquisition performance is shaped not only by hardware capabilities but also by the interplay between system design and signal characteristics.

**Figure 5:**
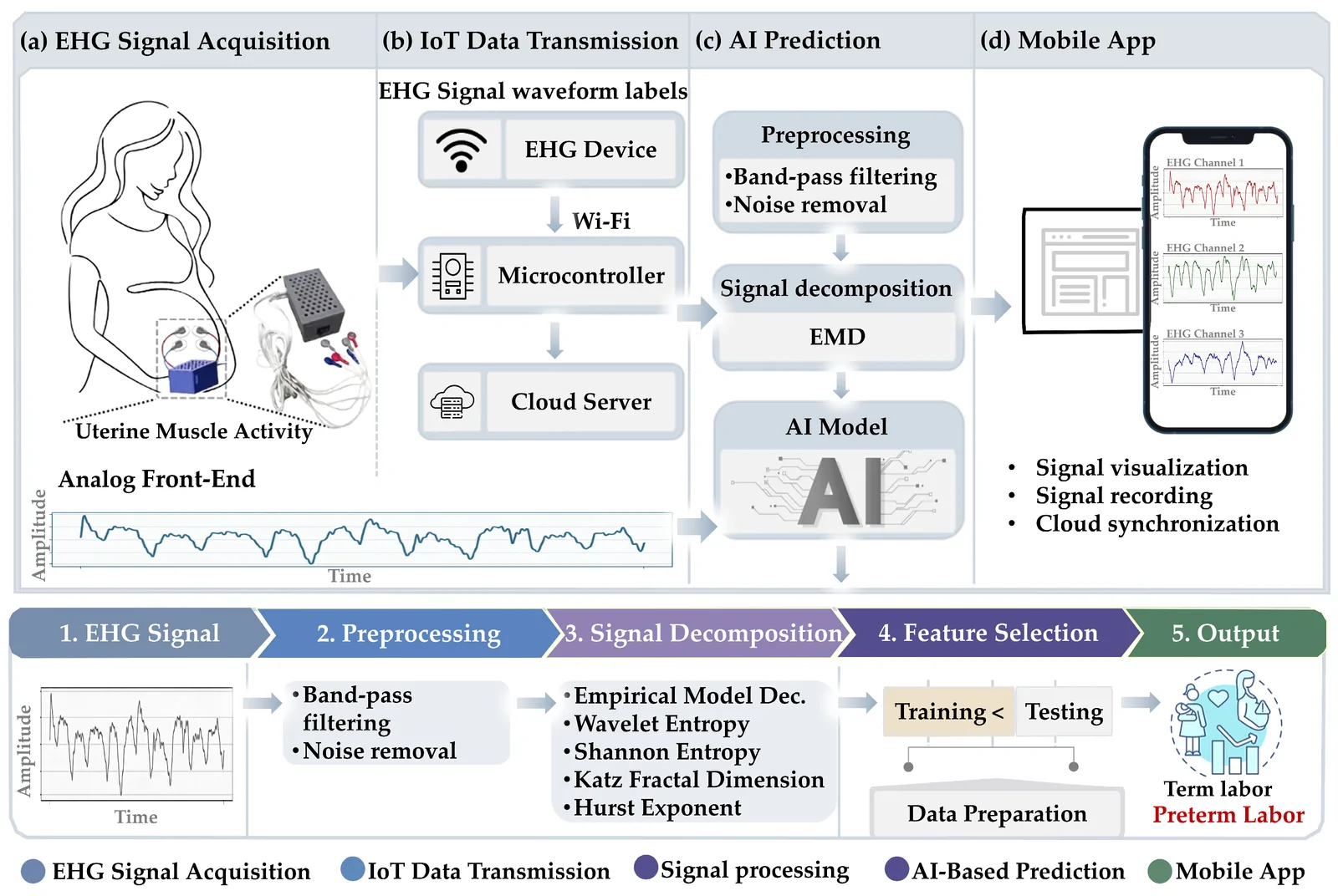
EHG-based wearable labor monitoring and prediction system. (**a**) EHG signal acquisition using a wearable electrode array and analog front-end; (**b**) IoT-based data transmission through a microcontroller and cloud server; (**c**) AI-based prediction including preprocessing, empirical mode decomposition, and machine learning analysis; and (**d**) mobile application for real-time visualization and recording. The lower panel represents the signal processing pipeline from EHG acquisition to feature extraction and classification, generating predictions of term or preterm labor (Author-developed schematic synthesized based on [75,85]).

Synthesizing architectural elements from recent EHG research [75,85], a schematic overview of the signal conditioning, artifact suppression, feature extraction, and machine learning-based prediction models for contraction detection and labor prediction is shown in Figure 5 [26,36,74,75,76,85]. Although these advanced analytical approaches demonstrate considerable potential, their effectiveness is ultimately constrained by the inherent signal variability and noise present in wearable environments [50,86]. This highlights that increasing algorithmic complexity alone cannot compensate for the absence of reliable input signals.

At the system level, the output layer facilitates real-time visualization, data storage, and clinical decision support [50,75,76]. However, inconsistencies in signal quality and the absence of standardized system integration undermine interpretability and clinical trust, particularly in ambulatory environments. As shown in Figure 4 and Figure 5, current system architectures lack sufficient unification. At the processing level, adaptive signal processing and data-driven approaches including machine learning and advanced feature extraction are increasingly employed to enhance contraction detection and labor prediction [36,74,85]. Despite these advancements, their performance remains constrained by signal quality and noise, which limit robustness in wearable applications. The literature therefore advocates for a transition toward modular and adaptive processing frameworks that emphasize reliability over algorithmic complexity. As shown in Figure 5, system architectures are typically structured into acquisition, processing, and interface layers [74,75,85].

Although these architectures are often modular, they typically lack comprehensive integration across acquisition, processing, and interface layers. This limited cross-layer integration constrains overall system performance, as enhancements in processing algorithms or hardware components do not consistently yield reliable clinical outcomes. In practice, insufficient coordination among electrode topology, signal acquisition, and processing frameworks results in compounded errors, reduced robustness, and limited generalizability in real-world settings. This limitation remains a significant challenge in the development of current EHG systems, where design decisions are frequently made in isolation rather than within an integrated framework. To address these challenges, the development of reliable wearable EHG systems necessitates coordinated considerations of sensing, processing, and system architecture. Such an integrated approach is essential to ensure that analytical performance is aligned with signal reliability and clinical applicability.

#### 4.2.3. Wearable Usability Assessment

Usability assessment is a critical factor influencing the clinical adoption of wearable EHG systems, with electrode adherence, patient comfort, and setup time identified as primary barriers in uterine monitoring and other biopotential systems [7,8,22,87]. Accordingly, practical usability represents a distinct component of clinical feasibility beyond the ability of the system to acquire an interpretable EHG signal. However, these factors are frequently evaluated in isolation, lacking systematic integration with signal quality and overall system performance metrics. Electrode adherence, in particular, is directly associated with signal continuity and artifact prevalence, as inadequate contact or motion can result in intermittent data loss and increased noise [1,10,26]. Although standardized evaluation methods, such as detachment scales and signal quality monitoring, have been proposed [7,8], their inconsistent use across studies hampers meaningful comparison and the generalizability of findings. Current evaluation protocols are often embedded within clinical implementation studies that combine noninvasive fetal ECG and EHG monitoring [17] or within comparative analyses against standard monitoring [8,10]. These methodologies typically integrate objective signal indicators with observational data collected during maternal movement. However, these approaches remain insufficiently linked to electrode topology and system design, despite the influence of these factors on signal reliability [1,10,26].

Patient and user experience has been evaluated using structured questionnaires incorporating continuous and ordinal rating scales together with preference questions and open-ended feedback [7,22]. In Thijssen et al. [7], patient experience was assessed using a 0–100 continuous scale, where lower scores indicated less bother. EHG received a median score of 2.1 (IQR 0.0–5.2) for application, compared with 10.0 (2.1–27.6) for TOCO and 30.6 (8.0–53.1) for IUPC. For presence during labor, EHG also received a median score of 2.1 (0.0–6.5), compared with 42.0 (10.8–58.7) for TOCO and 8.3 (1.0–47.0) for IUPC. Overall, 80.8% of the participating women preferred EHG over the other two monitoring methods. Healthcare professionals evaluated several aspects of EHG use using 5-point ordinal scales [7]. However, the available evidence does not provide a direct comparison of patient comfort across different EHG electrode counts, because electrode count was not evaluated as an independent usability factor. Such assessments provide complementary information on comfort, usability, and practical barriers that may not be captured by technical performance measures alone [7,22,87]. Incorporating posture-related factors, such as semi-recumbent and lateral positioning, may enhance the contextual interpretation of EHG signals [88]. However, the relationship between user comfort and long-term signal stability remains underexplored, thereby limiting the development of informed design considerations. Setup time and subsequent monitoring workload are important considerations for clinical usability. Although some studies report simplified EHG application or reduced setup burden, quantitative comparisons of setup duration across different EHG configurations remain limited [5,8,10,22]. In Thijssen et al. [7], 60.4% of nurses considered EHG more time-consuming than TOCO, with initial skin preparation and impedance assessment contributing to the workflow burden. However, during a 2-h monitoring period, EHG required approximately 1 adjustment on average, compared with 2 repositioning interventions for TOCO because of signal loss [7]. Thus, greater initial EHG setup effort may be partly offset by fewer subsequent interventions during monitoring. Although studies typically assess setup time using standardized timing protocols, reapplication events, such as belt repositioning or electrode readjustment, are reported as additional workflow burdens that influence clinical usability. Although wearable systems are designed to reduce setup complexity, repeated adjustments due to electrode displacement or suboptimal signal quality can introduce further workflow challenges. Clinician assessments of setup ease and signal interpretability indicate that usability is more closely associated with system reliability than with deployment simplicity alone [8,10,26].

Usability is an important consideration for wearable EHG systems, particularly with respect to electrode adherence, patient comfort, and setup time [1,5,72,89]. Practical usability therefore represents a distinct component of clinical feasibility beyond the ability to acquire an interpretable EHG signal. The findings presented in Section 4 highlight that current EHG system designs are limited by insufficient integration among electrode topology, signal acquisition, and processing frameworks. By synthesizing these interrelated factors, this study provides a coherent set of design guidelines for wearable EHG systems.

## 5. Discussion

### 5.1. Clinical Feasibility

Reliable acquisition of uterine electrical activity represents an essential technical prerequisite for wearable EHG monitoring, but does not, by itself, establish clinical feasibility. EHG records bioelectrical activity associated with myometrial activation rather than directly measuring the mechanical consequences of uterine contraction; therefore, the physiological relevance of the recorded signal and its relationship to uterine function and the intended clinical application must also be considered. In this review, clinical feasibility is considered more broadly in relation to practical implementation, patient-related factors, acquisition reliability, user acceptance, and compatibility with clinical workflow. Open-access datasets and prospective studies provide evidence that EHG can reliably capture uterine electrical activity across specific obstetric populations and monitoring conditions [3,56,65,90]. Beyond labor-related applications, individual studies have investigated EHG for monitoring changes associated with cervical dilation and for characterizing uterine electrical activity in nonpregnant physiological states [71,91]. These findings indicate potential areas for further investigation but should not be interpreted as establishing clinical utility for fertility or other reproductive applications. As noted in Section 4, the electrode–density ranges used in this review represent an author-developed descriptive categorization based on the configurations reported in the reviewed literature and should not be interpreted as standardized or clinically validated thresholds. The literature highlights regulatory compliance, signal robustness, and user acceptance as critical determinants of successful clinical implementation. Compliance with relevant standards and guidance, including IEC 60601-1 [80] for basic safety and essential performance, ISO 14971:2019 [81] for risk management, ISO 14155:2020 [92], and applicable ethical guidelines [93] for clinical investigations, can support the safety and risk assessment of wearable EHG systems [15,81]. However, such compliance does not by itself establish clinical safety or effectiveness. Notably, prior studies have reported no significant electromagnetic interference during continuous monitoring [10,75,85]. However, several reports emphasize that regulatory compliance alone does not guarantee clinical usability, particularly in complex and high-mobility environments such as labor wards.

Electrode material properties, including flexibility, adhesion, and stability at the electrode–skin interface, can influence signal quality and user comfort [62,93]. However, Section 4.2 highlights that material selection alone is insufficient to guarantee reliable performance under real-world clinical conditions. Factors such as motion artifacts, perspiration, and anatomical variability continue to compromise signal integrity.

Similarly, signal-based metrics including RMS and entropy-derived features have shown promise in distinguishing labor outcomes [1,67,76,94,95]. However, their robustness may be affected by noise, motion artifacts, and inter-patient variability, thereby limiting their generalizability across diverse clinical populations. These limitations reflect the broader issue identified in Section 4: the lack of consistent integration among electrode topology, signal acquisition, and processing, resulting in fragmented system performance. Furthermore, inadequate consideration of usability factors such as patient comfort, workflow compatibility, and clinician interpretability remains a significant barrier to clinical adoption [75,80]. In this context, electrode configuration emerges as a major determinant of clinical implementation. Evidence suggests that a medium to high electrode density may offer practical usability for wearable EHG design, although further validation is required across diverse patient populations and monitoring scenarios. This range aligns with the design principles established in Section 4, which emphasize the need for coordinated optimization of sensing, processing, and usability. Ultimately, effective clinical deployment depends on integrated design rather than on isolated improvements in hardware or algorithms.

### 5.2. Measurement Approaches in the Literature

Because biopotential-monitoring technologies measure different physiological electrical activities and serve distinct clinical objectives, their modality-specific diagnostic or clinical performance cannot be extrapolated to EHG. Related EHG literature addressing electrode configuration, wearable implementation, and acquisition considerations provides additional context for these technical considerations [25,37,73].

Measurement protocols typically involve continuous EHG acquisition during routine labor, often under varying maternal postures, to evaluate posture-dependent variability in uterine electrical activity [53,85]. Previous research has demonstrated that real-time monitoring, enhanced by advanced filtering and adaptive noise suppression, facilitated evaluation of signal stability, motion artifacts, and baseline drift in clinical settings [17,25,38,77]. As discussed in Section 4, the effectiveness of these approaches remains highly contingent on electrode stability, topology, and resistance to motion artifacts, which can limit consistency across conditions.

The literature further emphasizes usability-related factors, including setup time, electrode adherence, maternal comfort, and clinician interpretability, as essential for successful clinical deployment [15,55]. However, these factors are frequently evaluated independently of signal performance, despite their direct impact on data quality and workflow integration. This fragmentation remains a key limitation of current measurement methodologies, as they insufficiently integrate technical and user-centered considerations.

Overall, existing measurement approaches are constrained by variable acquisition conditions, electrode placement, motion artifacts, electrode–skin impedance, and stability. Future studies should standardize acquisition practices and incorporate usability and signal-quality evaluations to improve comparability and support clinical validation of wearable EHG systems.

Despite the comprehensive scope of this study, several limitations must be acknowledged. First, substantial heterogeneity across the included studies, in terms of study populations, electrode configurations, monitoring devices, signal acquisition protocols, outcome definitions, and study designs, limits direct comparison and precludes formal meta-analysis. In addition, direct head-to-head comparisons of different EHG systems and electrode configurations were limited, restricting conclusions regarding their relative performance. Second, the frequently reported use of 8–16 electrodes represents a practical range derived from the existing literature rather than a universally validated optimum, highlighting the need for standardized benchmarking and prospective clinical validation. Because no formal risk-of-bias or evidence-certainty assessment was performed, this literature-derived range should be interpreted as descriptive rather than quality-weighted evidence. Third, potential publication and retrieval biases remain a concern because the search strategy may not have captured all relevant literature, particularly material not indexed in the searched sources. Fourth, no formal systematic review protocol was prepared or prospectively registered before the review was conducted, which may limit the transparency and reproducibility of the review procedures. Finally, as this review synthesizes a system-level framework from the existing literature rather than presenting a validated prototype, the proposed guidelines should be regarded as a foundation for future empirical and clinical validation.

## 6. Conclusions

EHG represents a noninvasive modality for characterizing myometrial excitation and labor dynamics. This study critically examined the advancements in EHG systems from fragmented components toward the integrated, unified frameworks necessary for clinical translation. A key finding of this review is that configurations employing approximately 8–16 electrodes emerge from the reviewed literature as a practical range for balancing spatial sampling and implementation complexity. This range should be interpreted as a literature-derived indication rather than a validated optimum. Different monitoring objectives may require different levels of electrode complexity; therefore, electrode count and arrangement should be selected according to the intended application, required spatial information, and clinical setting.

Despite notable advances in flexible, modular, and real-time EHG systems, significant barriers to widespread clinical adoption persist. The study findings highlighted the insufficiency of incremental improvements in hardware or isolated signal processing techniques. Instead, coordinated optimization across the entire acquisition-to-analysis pipeline is essential. Notably, the absence of standardized electrode placement strategies, variability in signal quality under real-world conditions, and limited integration among system components continue to constrain both the reliability and generalizability of current approaches.

Future research should prioritize standardized benchmark datasets with clearly documented acquisition conditions, electrode configurations, participant characteristics, and clinically relevant outcomes to enable reproducible comparison across EHG studies. At minimum, cross-system benchmarking should report electrode-skin contact impedance, motion-artifact robustness, setup time, signal-to-noise ratio or an equivalent signal-quality metric, and clinically relevant endpoints using standardized definitions. Further development of motion-robust dry and textile electrode interfaces is needed to support reliable and comfortable prolonged monitoring under ambulatory conditions. Multimodal approaches combining EHG with complementary signals, such as noninvasive fetal ECG, may provide a more comprehensive assessment of maternal–fetal status while enabling shared wearable acquisition platforms. In parallel, data-efficient and interpretable machine-learning methods should be investigated to address limited labeled datasets and improve the transparency of clinically relevant predictions. Ultimately, prospective multicenter studies across diverse populations and clinical settings are required to establish generalizability, reproducibility, and clinical feasibility before widespread implementation.

## Figures and Tables

**Table 1:** Overview of wearable EHG systems and transferable biopotential-monitoring technologies, including electrode count and placement, signal acquisition characteristics, and clinical applications across monitoring settings.

Study	Evidence Type	Electrode		Signal Acquisition	Clinical Application
		Count	Placement		
Cohen et al. [14]	Transferable technical evidence	5	P1	Usable signal acquisition	Hospital (intrapartum monitoring)
Verdurmen et al. [39]	Transferable technical evidence	8	P1 & P2	Usable signal acquisition	Hospital/Clinical research setting
Hill et al. [40]	Direct EHG evidence	5	P1	Reported signal quality	Hospital (fetal monitoring)
Nichting et al. [41]	Transferable technical evidence	6	P1	Reported signal quality	Hospital/Academic medical center
Maria W. E. et al. [5]	Direct EHG evidence	6	P1	Usable signal acquisition	Hospital (labor ward)
Thijssen [42]	Direct EHG evidence	3–6	P1	Usable signal acquisition	Hospital/Clinical implementation
Boszko et al. [43]	Transferable technical evidence	12	P1 & P3	Reported signal quality	Hospital/Ambulatory clinic
Aggarwal and Wei [44]	Transferable technical evidence	2–6	P1	Reported signal quality	Clinic/Home-care research
Tsukada et al. [45]	Transferable technical evidence	Not reported	P3	Reported signal quality	Clinic/Ambulatory health facility
Paul et al. [46]	Transferable technical evidence	7	P3	Reported signal quality	Clinic/Research laboratory
Achilli et al. [47]	Transferable technical evidence	2	P3	Reported signal quality	Clinic/Wearable health monitoring
Acar et al. [48]	Transferable technical evidence	Not reported	Not reported	Usable signal acquisition	Clinic/Ambulatory care
Steinberg et al. [49]	Transferable technical evidence	3	P3	Reported signal quality	Clinic/Telehealth systems
Zheng et al. [50]	Transferable technical evidence	8	Not reported	Reported signal quality	Clinic/Home-care setting
Song and Lee [51]	Transferable technical evidence	2	P3	Reported signal quality	Clinic/Medical device material reference

**Table 2:** Summary of wearable EHG systems and transferable wearable biopotential-monitoring technologies, including evidence type, materials, electrode topology, user acceptance, and reported system limitations across studies.

Study	Evidence Type	Material	Electrode Topology	User Acceptance	Key Limitations
**Patch**					
Cohen et al. [14]	Transferable technical evidence	Not specified	Distributed	Well tolerated	Susceptibility to motion artifacts and signal degradation.
Verdurmen et al. [39]	Transferable technical evidence	Not specified	Distributed	Well tolerated	Signal variability due to fetal movement, maternal tissue, and noise.
Hill et al. [40]	Direct EHG evidence	Not specified	Distributed	Comfortable	Limited sensitivity during early labor.
Nichting et al. [41]	Transferable technical evidence	Not specified	Distributed	Well tolerated	Limited validation and sensitivity to fetal movement.
Maria W. E. et al. [5]	Direct EHG evidence	Not specified	Distributed	Comfortable	Signal instability due to motion and technical artifacts.
Thijssen [42]	Direct EHG evidence	Not specified	Distributed	Well tolerated	Sensitivity to noise, motion artifacts, and electrode placement variability.
Boszko et al. [43]	Transferable technical evidence	Not specified	Distributed	Well tolerated	EHG-specific clinical performance not evaluated.
Aggarwal and Wei [44]	Transferable technical evidence	Not specified	Distributed and Square grid	Well tolerated	Low-amplitude signals with susceptibility to interference and placement variability.
**Textile/Wearable**					
Aggarwal and Wei [44]	Transferable technical evidence	Nylon fabric, woven, nonwoven, and knitted materials/fabrics	Distributed and Square grid	Comfortable	Maternal ECG interference and placement-dependent variability.
Tsukada et al. [45]	Transferable technical evidence	Woven from nano-fibers	Placed inside the undershirt	Comfortable	Motion artifacts and unstable skin contact.
Paul et al. [46]	Transferable technical evidence	Stencil-printed carbon-loaded rubber, woven textile, thin layer/silver polymer	Distributed on the upper body	Comfortable	Positional mismatch, baseline drift, and motion artifacts.
Achilli et al. [47]	Transferable technical evidence	Polymer and a conductive ink	Not reported	Comfortable	High skin impedance and dry-condition noise susceptibility.
Acar et al. [48]	Transferable technical evidence	Conductive textile, fabrics and fibers, woven	Not reported	Comfortable	High impedance, motion artifacts, and unstable skin contact.
Steinberg et al. [49]	Transferable technical evidence	Woven wire	Distributed on the upper body	Well tolerated	Limited validation and uncertain extreme-body-weight performance.
Zheng et al. [50]	Transferable technical evidence	Woven with cotton and lycra	Distributed on the upper body	Comfortable	Baseline drift due to impedance and pressure variability.
Song and Lee [51]	Transferable technical evidence	Nylon and spandex yarns	Distributed on the upper body	Comfortable	Unstable skin contacts and motion-related signal variability.

Note: User acceptance categories were synthesized from qualitative usability descriptions reported in the cited studies. “Well tolerated” refers to systems that were easy to apply, clinically acceptable, minimally inconvenient, or suitable for routine monitoring and practical use [14,39,41,42,43,44,49]. “Comfortable” refers to systems associated with comfort, reduced skin irritation or discomfort, simplified placement, flexibility, breathability, convenience, or suitability for prolonged, ambulatory, or home-based monitoring [5,40,44,45,46,47,48,50,51]. These classifications were harmonized for comparative purposes and should not be interpreted as standardized or directly comparable quantitative usability outcomes across studies.

**Table 3:** Configuration-level comparison of reported EHG electrode arrangements and supporting electrodes, highlighting differences in electrode count, associated advantages, and limitations.

Electrode Count				Electrode Topology									Advantages	Limitations
	V	H	D	S_G_	D_I_	S_S_	R_G_	Supporting Electrode						
								R	G	R&G	DRL	CMR		
2 [18]	✓									✓			Simple electrode setup	Limited spatial information of uterine electrical activity.
		✓								✓				
3 [18]					✓					✓			Simple topology providing slightly more information than two electrodes.	Still provides limited spatial resolution for uterine activity analysis.
4 [18,58]	✓							✓					Enhances contraction detection and reliability while reducing electrode complexity.	Provides limited spatial information for uterine activity characterization.
			✓						✓					
			✓					✓			✓			
				✓				✓						
5 [18]	✓												Provides a balanced trade-off between system complexity and information content.	Still insufficient for detailed spatial mapping of uterine activity.
	✓									✓				
8 [3,18,57,58,61]	✓									✓			Multi-electrode topologies enable multichannel EHG acquisition, supporting spatial analysis of uterine activity and improving signal quality.	Increases system complexity and electrode placement sensitivity; remains susceptible to motion noise, inter-subject variability, and potential false detections.
			✓					✓				✓		
			✓					✓			✓			
						✓								
							✓							
16 [12,18,58,61,64,65,66,67,68,69,70]				✓									Large electrode arrays may provide more detailed spatial sampling and support spatial analysis of uterine electrical propagation and synchronization.	Requires complex setup and processing; prone to motion artifacts; may reduce clinical usability and patient comfort.
						✓				✓				

**Table 4:** Design justification for electrode density and spatial distribution, detailing the influence of electrode count and inter-electrode spacing on the detection of uterine electrical propagation.

Ref	Electrode Count	Inter-Electrode Spacing	Consideration to Determine the Count and Electrode Spacing
[65]	16 (4 × 4 grid)	17.5 mm	Spatial resolution and inter-channel crosstalk are balanced to preserve SNR for reliable propagation direction and velocity estimation.
[64]	16 + 2 R	17–25 mm	Motion artifacts and signal superposition are mitigated by iliac crest reference placement, ensuring stability and anatomical coverage.
[70]	16	Not specified	Electrode density is scaled to abdominal surface geometry to maintain uniform acquisition while limiting redundancy.
[57]	8 (4–2–2)	17.5 mm	Signal coherence is maintained across fundal initiation, mid-body propagation, and cervical termination regions.
[58]	6–16	17.5–25 mm	Propagation features are robustly extracted to facilitate graph-based coupling and phase synchronization analysis.
[59]	16	Not specified	Topology facilitates graph-theory-based analysis of connectivity and phase synchronization.
[66]	2	Not specified	Electrode spacing is adjusted to body habitus to maintain stable skin-electrode impedance.
[18]	Not specified	17.5–25 mm	Channel independence and signal fidelity are maintained while accommodating patient comfort constraints.
[61]	8	17.5 mm	Classification accuracy is optimized alongside the suppression of signal-overlap artifacts.
[67]	16	20 mm	Potential-field interpolation and phase-map resolution are enhanced for three-dimensional uterine modeling.
[68]	8	17.5 mm	High-resolution acquisition is balanced with stable extraction of root-mean-square (RMS), entropy, and velocity metrics.
[12]	8	20 mm	Inter-channel redundancy is reduced using correlation-based channel optimization.
[3]	8	17.5 mm	Data reproducibility and cross-study comparability are improved.
[69]	16	20 mm	Stable synchronization is maintained through controlled inter-electrode spacing.

## Data Availability

No primary datasets were generated for this study. All data supporting the findings were obtained from previously published articles, which are cited within the manuscript.

## Supplementary Materials

The following supporting information can be downloaded at: https://www.mdpi.com/article/10.3390/s26206539/s1, Table S1: Database-specific search queries; Table S2: Characteristics of the 91 publications included in the evidence synthesis; Table S3: PRISMA_2020_checklist.

## Abbreviations

The following abbreviations are used in this manuscript:

WHO	World Health Organization
UA	Uterine activity
UCs	Uterine contractions
EHG	Electrohysterography
TOCO	Tocodynamometry
IUPC	Intrauterine pressure catheters
ECG	Electrocardiography
AI	Artificial intelligence
IEEE	Institute of Electrical and Electronics Engineers
ICA	Independent component analysis
DRL	Driven right leg
CMRR	Common-mode rejection ratio
CMR	Common-mode reference
IEC	International Electrotechnical Commission
ISO	International Organization for Standardization
RMS	Root-mean-square
SNR	Signal-to-noise ratio
